# Development of the Healthy Women Intervention to Increase Women’s Engagement in Medication Treatment for Opioid Use Disorder: Mixed Methods, User-Centered Design Approach

**DOI:** 10.2196/85195

**Published:** 2026-03-31

**Authors:** Dawn E Sugarman, Emily A Levine, Callie L Wang, Francesca M Korte, Nicole A Barbaro, Roger D Weiss, Lisa A Marsch, Aimee NC Campbell, Shelly F Greenfield

**Affiliations:** 1 Division of Alcohol, Drugs, and Addiction, McLean Hospital, 115 Mill St, Belmont, MA, 02478, United States, 1 6178553650; 2 Department of Psychiatry, Harvard Medical School, Boston, MA, United States; 3 Division of Women's Mental Health, McLean Hospital, Belmont, MA, United States; 4 Department of Psychological Sciences, University of Vermont, Burlington, VT, United States; 5 Department of Psychology, University of Central Florida, Orlando, FL, United States; 6 Department of Public Health and Health Sciences, Northeastern University, Boston, MA, United States; 7 Geisel School of Medicine, Dartmouth College, Hanover, NH, United States; 8 Department of Psychiatry, Columbia University Irving Medical Center and New York State Psychiatric Institute, New York, NY, United States

**Keywords:** internet intervention, web-based intervention, opioid use disorder, mobile apps, women, digital technology, user-centered design

## Abstract

**Background:**

Rates of opioid use disorder (OUD) have increased among women over the past 2 decades. Medication treatment for opioid use disorder (MOUD) is effective but underused. Gender-specific treatments for women have been associated with improved substance use outcomes. However, these treatments have not specifically targeted women’s engagement in MOUD, and the impact of existing gender-specific treatments is restricted by in-person delivery.

**Objective:**

The aim of this study was to develop a digital intervention to feasibly deliver gender-specific care that addresses the individualized needs of women with OUD to increase engagement in MOUD.

**Methods:**

A mixed methods, user-centered design approach was used to inform the development of a digital intervention. In phase 1, qualitative interviews were conducted with women with lived experience of OUD (n=20) and providers who treat women with OUD (n=8). Interviews were recorded, transcribed, and coded for themes. In addition, a larger sample of treatment providers (n=55) completed an online survey to further inform the content of the digital intervention. Phase 2 consisted of designing, beta-testing (n=5), and refining the intervention.

**Results:**

The age of women with lived experience ranged from 21 to 59 (mean 38.5, SD 9.4) years; 63% (5/8) of providers interviewed were female participants. The qualitative interview data from women with lived experience and providers were grouped into 6 thematic categories: 3 treatment-related (1) barriers to treatment, (2) facilitators to successful recovery, and (3) important issues to address in treatment, and 3 technology-related (4) positives of using technology as part of treatment, (5) suggested technology features, and (6) barriers to using technology. Across the treatment-related categories, several themes touched on women-specific factors including family responsibilities, abusive partners, stigma, and motivation for treatment (eg, pregnancy). The technology-related categories provided information for designing the features of the intervention, as well as revealing barriers to technology use, which could be helpful in developing implementation strategies. Provider survey participants were primarily female participants (40/55, 73%), with a mean age of 42.5 (SD 12.5) years. Survey data provided additional information on barriers to treatment and suggested technology features. Based on these data and preliminary work, the *Healthy Women* intervention was created. Minor edits to content and visual design were made in the beta-testing phase. The final version includes a web-based component with 6 topic modules and a mobile component. Topics in the web-based component are presented through infographics, text, videos, and interactive questions. The mobile component includes daily motivational messages, skills practice activities (2/wk), weekly check-ins, and resources (always available).

**Conclusions:**

Important themes and suggested features from women with lived experience and providers were incorporated into a digital intervention for women with OUD. Data on feasibility, satisfaction, and engagement with the *Healthy Women* intervention are currently being collected in phase 3, a pilot randomized controlled trial.

## Introduction

### Background

In 2024, 2.48 million women had a past-year opioid use disorder (OUD) [1]. Although rates of substance use disorders (SUDs) overall are higher in men than in women, rates of OUD are equivalent, and the reverse pattern has emerged for adolescents, with higher rates of OUD in girls compared to boys [1]. Medication treatment for opioid use disorder (MOUD) is effective in reducing opioid use and the risk of overdose, but retention rates are low, particularly during the early phase of treatment [2,3]. Among adults with OUD, females are less likely to receive MOUD compared to males [1]. Therefore, it is critical to develop specific sustainable strategies to engage and retain women with OUD in MOUD treatment.

Gender-specific SUD treatments address factors that are more prevalent in women, which may affect their treatment outcomes (eg, exposure to trauma, co-occurring psychiatric disorders, relationships with children and intimate partners, and physical health issues) and are associated with increased engagement, greater satisfaction, and improved outcomes [4-7]. However, these treatments have not specifically targeted women’s retention in MOUD treatment, and the impact of existing gender-specific treatment is restricted by in-person delivery. Fewer women relative to men in mixed-gender settings can be an additional barrier to implementing gender-specific treatments in substance use treatment programs [8].

Technology-based interventions are effective in encouraging treatment engagement [9-11]. Smartphone apps, in particular, are emerging as a feasible and desirable way to integrate frequent support into patient care, with recent work demonstrating high perceived usefulness, helpfulness, and enthusiasm about various apps from OUD patients and providers alike [12-16]. However, much of the current work in this area has used mixed-gender participant populations and has not specifically focused on women’s experiences [9-11,13,14,16,17].

Findings from a recent review of digital interventions to support women in treatment for OUD showed that most of the existing technology in this area involves telemedicine (ie, virtual visits) rather than asynchronous digital interventions [18]. Since recent work has found that one of the biggest barriers for women in OUD treatment is time, it is crucial to fill the gap in research around self-guided recovery-focused technology [19]. The few studies that examine digital interventions created for women with OUD have targeted highly specific populations, such as women in rural areas mandated to outpatient treatment [20] and American Indian or Alaska Native women [21], highlighting the need for interventions adaptable to less specific patient populations.

### Prior Work

To examine the feasibility of creating a gender-specific digital intervention for women with SUDs, we adapted material from the Women’s Recovery Group (WRG) [5,6,22] to a web-based format. The WRG is an evidence-based, women-focused, single-gender group therapy for women with SUDs [5,6,22]. This 12-session group is based on a cognitive-behavioral approach and includes gender-specific content and relapse prevention skills. In a series of pilot studies [23], we adapted material from modules of the WRG that are of key relevance to women with SUDs into a web-based format within a user-friendly design. Interactive questions were included to increase engagement with the digital intervention. Next, the intervention was tested with 30 women receiving mixed-gender inpatient care in McLean Hospital’s Alcohol, Drug, and Addiction Treatment Program (ADATP). Results revealed a high level of satisfaction with the intervention. Based on participant feedback, the intervention was refined, including the addition of material from two additional WRG modules. These changes directly addressed participants’ interest in receiving more information on trauma and sexual abuse, as well as self-care in recovery. We then tested the intervention with 60 women receiving inpatient, partial hospitalization, or outpatient levels of care [23] and again found a high level of satisfaction with the intervention. Satisfaction scores did not differ by level of care, prior treatment experience, or age. These data support the feasibility of a gender-specific digital intervention for SUDs, show that women were highly satisfied with this type of program, and provide a prototype of the intervention for the current study.

### Objective

Building on our previous work, the aim of the study was to gather data from treatment providers and women with lived experience of OUD to inform the development of a digital intervention to feasibly deliver gender-specific care that addresses the individualized needs of women with OUD, thereby increasing engagement in MOUD treatment.

## Methods

### Overview

**Figure 1.**
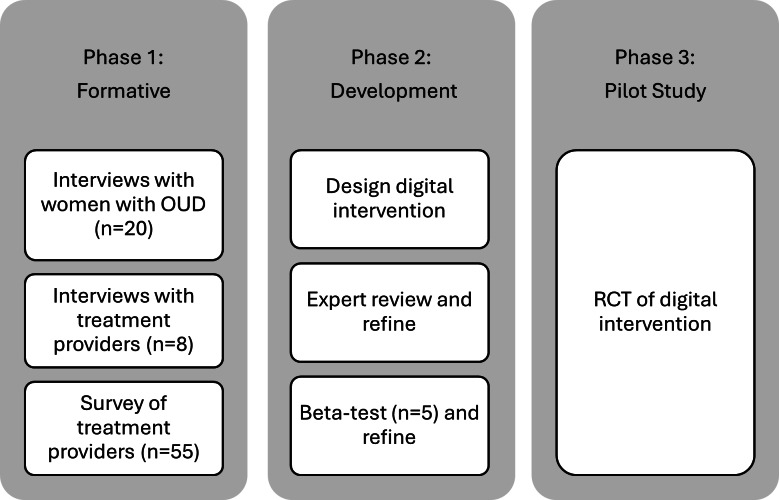
Overview of study phases. OUD: opioid use disorder; RCT: randomized controlled trial.

A mixed methods approach was used to inform the development of the digital intervention. Figure 1 outlines the 3 phases of the study. The development process incorporated principles of user-centered design based on the Discover, Design and Build, and Test (DDBT) framework [24]. In phase 1 (discover), we conducted formative work by interviewing women with lived experience as well as providers who treat women with OUD. To further elucidate the provider perspective, we also administered a self-report survey to a larger sample of treatment providers. Phase 2 (design or build) was a modified version of the steps outlined in the DDBT framework, as we were building off an existing prototype that had already undergone 2 rounds of usability testing [23]. In this phase, we further adapted the existing prototype based on feedback from the formative phase. Next, there were two rounds of refinement: (1) following expert review and (2) after beta-testing phases 1 and 2 are reported in this paper. Phase 3 (test), a pilot randomized controlled trial of the intervention, is currently underway. 

### Ethical Considerations

The Mass General Brigham Institutional Review Board (IRB) reviewed and approved the protocols for this study (phase 1: protocols #2019P003747 and #2022P001902; phase 2: protocol #2023P000495) and waived the requirement for written consent for phase 1 studies. Participants were apprised of the study’s objectives, content, and associated risks and benefits through online study fact sheets. Eligible participants expressed their willingness to participate by clicking on a checkbox next to the statement “I agree to participate in this research.” For phase 2, participants completed electronic informed consent after reading through the IRB-approved consent form with a research staff member available to answer any questions, explain the study procedures, and review the risks and benefits of the study. REDCap (Research Electronic Data Capture) was used to obtain electronic informed consent. To protect privacy and confidentiality, participants were reminded at the beginning of the qualitative interviews to avoid disclosing identifying information. If any identifying information was inadvertently disclosed, it was removed from interview transcripts. No information concerning individual cases is referred to in public materials in such a way as to identify individuals.

In phase 1, provider participants who completed qualitative interviews received a US $25 gift card for their participation. The first 16 women with lived experience participants received a US $25 gift card. Partway through the study, there was recognition that due to the COVID-19 pandemic, individuals had increased economic needs. After discussion with clinicians in community clinics and the IRB, the women with lived experience participant payment was increased to a US $50 gift card; the final four participants received the US $50 gift card. Provider participants who completed the survey had the option to enter a raffle for a chance to win a US $50 gift card. In phase 2, participants received US $70 for the baseline visit.

### Phase 1: Formative Research

#### Participants and Procedures

##### Qualitative Interviews

Women with lived experience (N=20) were recruited from inpatient (n=10), residential (n=4), and outpatient (n=6) treatment programs in the greater Boston area via referral from clinical providers or self-referral from study advertisements. Women were eligible if they were at least 18 years old and receiving care for a current OUD. Women were ineligible for the study if they were admitted to inpatient treatment involuntarily or had an acute psychiatric or medical condition or cognitive impairment that would hinder their ability to complete study procedures. Women with lived experience ranged from 21 to 59 (mean 38.5, SD 9.4) years of age and endorsed several co-occurring mental health problems in the past year, the most common being anxiety (n=20, 100%), depression (n=19, 95%), and posttraumatic stress disorder (n=16, 80%). Nearly all (n=18, 90%) had tried some form of MOUD in their lifetime, and 65% (n=13) were currently taking MOUD at the time of the interview.

Treatment provider participants (N=8) were invited to participate in the study if they provided clinical services to individuals with OUD in the McLean Hospital’s ADATP continuum.

Once enrolled in the study, all participants completed semistructured interviews with the principal investigator (DES). Interviews were conducted either in-person at McLean Hospital or remotely over Zoom. Interviews conducted with women with lived experience explored topics most important to them regarding their treatment, treatment barriers and facilitators, and their interest in using technology and/or mobile apps in their recovery (Multimedia Appendix 1). The women with lived experience also completed a web-based survey using REDCap. The survey included questions about participant demographics, substance use, and history of SUD treatment. Interviews conducted with treatment providers explored providers’ perspectives on women’s reasons for initiating MOUDs, barriers to maintaining sustained MOUD treatment, and the role of technology in helping women initiate MOUDs (Multimedia Appendix 2).

Interviews were conducted between January 2021 and December 2021. All interviews were audio-recorded and then transcribed. Transcripts were coded using inductive thematic analysis in NVivo (Lumivero) [25] in accordance with the 6 phases described by Braun and Clarke [26]. For the women with lived experience interviews, 2 coders (DES and CLW) independently reviewed the transcripts and determined initial codes. The codes were reviewed, and a master code list was defined and agreed upon by the 2 coders; next, they independently coded 5 of the transcripts. Interrater reliability was established (percent agreement across the 5 coded transcripts=99%), and CLW coded the remaining transcripts. Once complete, DES reviewed the coded transcripts and met with CLW for a final time to discuss and resolve discrepancies. Next, codes were examined, and related codes were clustered together into themes based on similarity.

A similar process was followed for coding the provider interviews; however, given the small number of transcripts, two coders (CLW and FMK) independently coded all the transcripts. DES met with the two coders to discuss and resolve discrepancies.

##### Provider Survey

To answer an online survey, treatment providers were recruited through relevant national organizations’ email listservs and snowball sampling via local treatment providers. Providers were eligible to enroll in the study if they were 18 years of age or older and had provided clinical services to women with OUD in the past year (as indicated by responses to self-reported screening questions). Providers who were eligible and agreed to participate (n=58) completed a brief self-report survey. Three of the 58 providers who agreed to participate in the study did not complete any of the survey questions. Therefore, the available sample for analysis consisted of 55 participants. Descriptive statistics were used to summarize data from the provider survey. 

The self-report survey was delivered via REDCap and was conducted between September 2022 and December 2022. The survey was developed specifically for this study and included questions on provider demographic characteristics and professional background, perceptions of barriers to treating women with OUD, frequency of advising women about MOUD, and ratings of the usefulness of different features and content of a digital intervention for women with OUD.

### Phase 2: Development

#### Beta-Testing: Participants and Procedures

The first 5 participants who enrolled in the pilot study and were assigned to the intervention condition served as the beta-testing group for the web-based component. Participants were recruited from McLean Hospital’s ADATP services continuum, the Mass General Brigham online research recruitment site (Rally), and from flyers posted throughout McLean Hospital. Women were included if they (1) were 18 years of age or older, (2) had a current OUD diagnosis (moderate or severe by DSM-5 criteria), (3) initiated MOUD in the past 30 days, and (4) were able to provide informed consent. Women were excluded if they (1) had an acute psychiatric or medical condition, or cognitive impairment that would impair the ability to complete study procedures (as determined by review of medical records and confirmation from clinical staff), or (2) were admitted to their current treatment episode on an involuntary status.

All 5 participants engaged in a “think aloud” process as they navigated through the web-based portion of the intervention, which occurred at the baseline visit for the pilot study. Specifically, they were asked to describe their experiences and any challenges they encountered. The research assistant took notes during this process. Participant comments were reviewed and discussed by members of the study team (DES, NAB, SFG, and RDW).

## Results

### Qualitative Interviews

#### Overview of Thematic Analysis

Demographic characteristics of the qualitative interview participants are reported in Table 1. Themes for the qualitative interviews with women with lived experience and treatment providers were reviewed for commonalities. There was considerable overlap in the themes that emerged from the 2 sets of qualitative interviews, given that the interview questions were similar. Thus, we grouped the themes from both sets of interviews into 6 common thematic categories, which followed the main topics of the interview questions. Three categories were related to treatment, and 3 were related to technology. Table 2 shows the 6 categories and associated themes from both the provider and women with lived experience interviews. Five categories were common across both sets of participant interviews: barriers to treatment, facilitators to successful recovery, positives of using technology as part of treatment, suggested technology features, and barriers to using technology. An additional category was relevant to the women with lived experience interviews only: important issues to address in treatment. The women with lived experience participants were specifically asked a question about important topics to address in treatment, whereas treatment providers were not asked this question.

**Table 1.** Demographic characteristics of qualitative interview participants.

Participants	Total
Women with lived experience (N=20)	
Age (y), mean (SD; range)	38.5 (9.4; 21-59)
Race^a^, n (%)	
White	15 (75)
Black or African American	1 (5)
American Indian or Alaskan Native	1 (5)
Other	1 (5)
Prefer not to answer	2 (10)
Ethnicity, n (%)	
Hispanic or Latina	5 (25)
Sexual orientation, n (%)	
Heterosexual or straight	14 (70)
Bisexual	3 (15)
Lesbian	1 (5)
Pansexual	1 (5)
Prefer not to answer	1 (5)
Marital status, n (%)	
Never married	11 (55)
Married	3 (15)
Separated	4 (20)
Divorced	2 (10)
Education, n (%)	
Less than high school	3 (15)
High school graduate or GED^b^	4 (20)
Some college	7 (35)
College graduate	6 (30)
Primary residence, n (%)	
Apartment/house	15 (75)
Homeless or shelter	4 (20)
Temporary housing	1 (5)
Living situation, n (%)	
Live alone	2 (10)
Live with parents or guardians	3 (15)
Live with extended family	5 (25)
Live with friends or roommates	4 (20)
Live with partner or significant other	5 (25)
Prefer not to answer	1 (5)
Occupational status, n (%)	
Unemployed	12 (60)
Disability	3 (15)
Employed part-time	2 (10)
Student	1 (5)
Work within the home	1 (5)
Prefer not to answer	1 (5)
Have children, n (%)	14 (70)
How many? mean (SD)	2.1 (1.3)
OUD^c^ treatment providers (N=8)	
Female, n (%)	5 (62.5)
Years working with OUD patients, mean (SD)	18.8 (10.4)
Discipline, n (%)	
Psychiatrist	7 (87.5)
Nurse (APRN^d^)	1 (12.5)

a Responses are not mutually exclusive.

b GED: general educational development.

c OUD: opioid use disorder.

d APRN: advanced practice registered nurse.

**Table 2.** Categories and themes from qualitative interviews.

Categories and themes	Examples codes	Discussed by	
		Women with lived experience (n=20)	Providers (n=8)
Barriers to treatment			
Co-occurring disorders	Depression Trauma Chronic pain Polysubstance use	✓	✓
Partners	Partner who uses substances Abusive partner	✓	✓
Negative beliefs			
Negative beliefs about SUD^a^ treatment	Belief that treatment does not work Dislike certain treatment modalities (eg, groups, telehealth)	✓	
Negative beliefs about MOUD^b^	Substituting one drug for another Short-term solution Stigma from others for being on MOUD Concerns MOUD use will affect future care	✓	✓
Personal factors or attitude	Lack of motivation and ambivalence Shame and embarrassment Guilt	✓	✓
MOUD-specific barriers	Concerns about tapering off Side effects or bad reaction Issues getting on the right dose Daily commitment (Methadone; Buprenorphine) Waiting period to initiate (Buprenorphine) Need to be abstinent to start (Vivitrol)	✓	✓
Family responsibilities	Childcare and caregiving responsibilities Pregnancy		✓
Facilitators to successful recovery			
Support from others	Mutual help groups Supportive friends and family Supportive partners	✓	✓
Motivation	Having accountability Commitment to treatment Factors motivating MOUD initiation (eg, withdrawal, craving, and pregnancy)	✓	✓
Structure	Having a schedule or routine	✓	
Coping skills	Engaging in healthy coping activities Avoiding negative influences	✓	
Mental health treatment	Getting treatment for co-occurring disorders	✓	✓
Education	Education about MOUD		✓
Important issues to address in treatment			
Co-occurring disorders	Trauma and abuse (sexual abuse, IPV^c^) Co-occurring mental health disorders Polysubstance use	✓	
Negative beliefs and emotions	Stigma Shame and guilt Grief	✓	
Social network	Peer support Being around positive influences	✓	
Woman-specific issues	Sexism Menstrual cycle Pregnancy and post-partum	✓	
Relationships	Partners Caregiving	✓	
Self-care	Forgiveness and self-love Honesty	✓	
Positives of using technology as part of treatment			
Availability	Easy to access Provides in the moment support	✓	✓
Connection with others	Helps with connectedness Easier to reach out to others for support through technology	✓	✓
Adjunct to treatment	Helps address co-occurring disorders Providers could encourage use Help with medication adherence	✓	✓
Accountability	Engaging with technology program regularly keeps people accountable to their recovery	✓	✓
Suggested technology features			
Reminders	Appointment or medication reminders Calendar	✓	✓
Tracking	Tracking substance use and cravings MOUD adherence Tracking mental health symptoms Tracking progress toward goals	✓	✓
Motivational supports	Motivational messages Rewards Personalized feedback Stories of people in recovery Journal	✓	
Educational information	Resources Location services to find meetings	✓	✓
Social support tools	Peer support Connecting with other women Connection with therapist Alerts to other people when relapsing	✓	✓
Skills practices	Meditation or mindfulness activities DBT^d^ skills	✓	
Barriers to using a technology			
Technological difficulties	No internet connection or no data Lack of access to phone Too difficult to use, not tech savvy Cost	✓	
Mental health or substance use	Actively using substances or relapse Mental health symptoms (eg, depression)	✓	
Lack of interest	Lose interest or forget Other priorities Lack of human connection	✓	
Provider issues	Liability concerns Burden on provider		✓

a SUD: substance use disorder.

b MOUD: medication treatment for opioid use disorder.

c IPV: intimate partner violence.

d DBT: dialectical behavior therapy.

#### Thematic Category 1: Barriers to Treatment

Participants identified several types of factors that impeded their ability to seek treatment and obtain MOUD. The 7 themes in this category encompass internal factors (eg, co-occurring disorders, negative beliefs, and attitudes) and external factors (eg, partners and family responsibilities). The theme of negative beliefs was split into two subthemes: negative beliefs about SUD treatment in general and negative beliefs specifically about MOUD. A separate theme of MOUD-specific barriers was included, which differed from negative beliefs about MOUD. Negative beliefs were often described as reasons people did not want to initiate MOUD (eg, “some of the support groups like Narcotics Anonymous have given messages to people that, ‘you’re just substituting one addiction for another.’” [provider]. MOUD-specific barriers tended to be concerns or issues expressed by people who wanted to take MOUD (eg, side effects or concerns about tapering off and dosing schedule); 1 participant described:

*I heard that it’s very hard to kick, to come off of it. So that’s one thing I’m kind of worried about*.

#### Thematic Category 2: Facilitators to Successful Recovery

In contrast to barriers, participants identified factors that were helpful to them in their recovery, including support from others, motivation, having structure, learning coping skills, getting mental health treatment for co-occurring disorders, and receiving education on MOUD.

Some themes that were identified included factors that could serve as both facilitators and barriers to treatment. For example, partners could act as a barrier to treatment if they used substances or were abusive, but partners who were supportive could serve as a facilitator. One provider described how partners can be a barrier to MOUD: “I’ve heard lots of different situations where their partner has sold their medication or their partner makes it difficult for them to get to their treatment appointments so they’re not able to fill their script.” Another provider described how partners can help with MOUD adherence: “Certainly partners that are supportive of them being on medication is a huge factor, especially if that partner is involved in monitoring the medication adherence in any meaningful way.” Pregnancy could also be a barrier to treatment, particularly in deciding to initiate MOUD, or it could facilitate treatment if it motivated women to enter treatment.

Both women with lived experience and providers identified system-level barriers and facilitators. Codes related to system-level barriers and facilitators are not included in the summary table (Table 2) as they did not directly inform the development of the digital intervention. Examples of system-level barriers identified by women with lived experience and providers included housing insecurity, legal issues, problems navigating the health care system, and a lack of beds available for women in SUD treatment programs. Examples of system-level facilitators included employment, case management, insurance coverage, and having a therapist or treatment provider who was caring and supportive.

#### Thematic Category 3: Important Issues to Address in Treatment

Many of the themes under this category were similar to some of the identified barriers to treatment, including co-occurring disorders, negative self-beliefs, women-specific issues, and relationships. Despite the overlap, these themes were retained under separate categories, as they can inform both the implementation of the intervention and its content. Participants also indicated that peer support and self-care topics, such as forgiveness, self-love, and honesty, were important to address in treatment. One woman with lived experience described self-care as being particularly relevant for women:

*...you’re not only harming yourself, but you’re also a mother and a caretaker, and you don’t get a lot of forgiveness, a lot of self-forgiving. I think we try so hard to be everyone to everyone, and…this sounds so weird to say, but I feel like it’s easier to forgive a man than it is to forgive a woman*.

#### Thematic Category 4: Positives of Using Technology as Part of Treatment

Themes in this category focused on the widespread availability of technology, the ability to easily connect with others, ways they could use digital programs with their treatment providers, and how technology-based programs could help keep them accountable to their recovery goals. Regarding accountability, 1 woman with lived experience stated:

*It’s kinda something to hold you accountable. Something to look forward to, and something that you know you’ve got to check in*.

#### Thematic Category 5: Suggested Technology Features

Participants reported several different types of features that could be helpful to include in a digital intervention for women with OUD. Suggested technology features were grouped into six themes: reminders, tracking, motivational supports, educational information, social support tools, and skills practices. With regard to tracking, there were several ways that participants thought this feature could be helpful, including monitoring substance use and cravings, MOUD adherence, mental health symptoms, and progress toward individual recovery goals. One participant described how the digital skills practice exercises could be especially helpful to individuals in recovery:

*That would be absolutely amazing [to have a skills practice feature]. Because as addicts…we forget things. And sometimes we go on autopilot, and we act on impulse. So if we had something like that to remind us, “This is how you cope, this is how you practice.” I think that would be excellent.*

#### Thematic Category 6: Barriers to Using Technology

Despite interest in using technology as part of treatment, women with lived experience identified some barriers to its use, including technological difficulties, lack of interest, and active substance use. Lack of access to mobile phones was one of the technological difficulties identified, particularly related to consistent access. As described by 1 woman with lived experience:

*Sometimes it comes down to—for somebody like me—it’s either food, shelter, sick, or paying my cell phone bill. My cell phone bill is going to be the last one, I’ll tell you that*.

Providers expressed concerns about liability and burden on the treatment provider. For example, 1 provider wondered:

*...an app that they could go login and either chat with each other, post something to some kind of discussion, would be really helpful…if I [the clinician] were involved in that it would be challenging because what if somebody posted something risky or safety wise or just privacy wise?*

### Provider Survey

Demographic characteristics of the survey sample are listed in Table 3. Provider survey participants predominantly identified as women and came from a range of disciplines, most commonly psychiatrists (13/55, 23.6%), other physicians (16/55, 29.1%), and psychologists (9/55, 16.4%). More than half of the samples were prescribers (30/55, 54.5%). Most providers (46/55, 83.6%) endorsed that they “usually” or “always” advise buprenorphine maintenance to women with OUD, whereas fewer providers endorsed “usually” or “always” advising naltrexone or methadone maintenance (16/55, 29.1% and 10/55, 18.2%, respectively).

**Table 3.** Demographic characteristics of the provider survey sample (n=55).

Demographic characteristics	Values
Age (y), mean (SD)	42.5 (12.5)
Gender identity, n (%)	
Woman	40 (72.7)
Man	14 (25.5)
Nonbinary or gender nonconforming or gender-queer	1 (1.8)
Race^a^, n (%)	
White	45 (81.8)
Asian	8 (14.5)
American Indian or Alaska Native	1 (1.8)
Multiracial	1 (1.8)
Prefer not to answer	1 (1.8)
Ethnicity, n (%)	
Hispanic or Latino	2 (3.6)
Discipline, n (%)	
Physician (other than psychiatrist)	16 (29.1)
Psychiatrist	13 (23.6)
Psychologist	9 (15.3)
Social worker	7 (11.9)
Nurse (RN^b^, APRN^c^, LPN^d^)	2 (3.6)
Other (recovery coach, mental health counselor, intern, student, community health worker)	8 (14.5)
Total years in practice, n (%)	
< 5 y	9 (16.4)
5-10 y	20 (36.4)
11-20 y	17 (30.9)
20+ y	9 (16.4)
Licensed prescriber, n (%)	30 (54.5)
Prescribed buprenorphine in the last 12 mo	28 (93.3)
Prescribed naltrexone in the last 12 mo	24 (80)
Referred any patients to an opioid treatment program for methadone treatment in last 12 mo, n (%)	30 (54.5)

a Responses are not mutually exclusive.

b RN: registered nurse.

c APRN: advanced practice registered nurse.

d LPN: licensed practical nurse.

More than half of the sample categorized the following as “major barriers” to treating women with OUD: housing insecurity (37/55, 67.3%), family responsibilities or lack of childcare (34/55, 61.8%), and lack of transportation (33/55, 60%) (Table 4). Participants also had the option to write in any additional barriers that were not listed. The most common write-in responses were stigma (n=7), comorbid mental health or medical issues (n=3), employment (n=3), and lack of a mobile phone (n=2).

**Table 4.** Provider-rated barriers to treating women with opioid use disorder.

Provider-rated barriers	Major barrier, n (%)	Minor barrier, n (%)	Not a barrier, n (%)
Patient housing insecurity	37 (67.3)	16 (29.1)	2 (3.6)
Patient lack of childcare or other family responsibilities	34 (61.8)	17 (30.9)	4 (7.3)
Patient lack of transportation	33 (60)	20 (36.4)	2 (3.6)
Patient denial or resistance	23 (41.8)	22 (40)	10 (18.2)
Lack of facilities or resources for treatment of OUD^a^, once identified	23 (41.8)	20 (36.4)	12 (21.8)
Patient inability to pay for treatment	20 (36.4)	15 (27.3	20 (36.4)
Patient negative beliefs about MOUD^b^	18 (32.7)	33 (60)	4 (7.3)
Partner or significant others interfering with patients’ treatment	17 (30.9)	33 (60)	5 (9.1)
State reporting laws and repercussions	16 (29.1)	19 (34.5)	20 (36.4)
Lack of or inadequate financial reimbursement for opioid screening, assessment, and counseling	16 (29.1)	17 (30.9)	20 (36.4)
Patient concerns about becoming addicted to MOUD	15 (27.3)	34 (61.8)	6 (10.9)
Limited training or experience in treating patients with OUD	11 (20)	10 (18.2)	34 (61.8)
Concern about patient confidentiality issues	7 (12.7)	27 (49.1)	21 (38.2)
Patient sensitivity to this topic (eg, fear of offending patients)^c^	1 (1.8)	24 (43.6)	29 (52.7)

a OUD: opioid use disorder.

b MOUD: medication treatment for opioid use disorder.

c Response missing from 1 participant.

Provider survey participants were given a list of potential features and content for a digital intervention and asked to rate how useful each one would be for women with OUD on a scale of 1 (not at all useful) to 5 (extremely useful). Mean ratings are reported in Table 5. Six features received a mean score of 4 or higher: (1) peer support, (2) appointment reminders, (3) connection with treatment providers, (4) crisis resources, (5) rewards, and (6) coping skills practice.

**Table 5.** Features or content of a digital intervention that would be most useful to women with opioid use disorder (n=55).

Features or content	Mean (SD)
Peer support	4.31 (0.88)
Appointment reminders	4.29 (0.79)
Connection with treatment provider	4.25 (0.80)
Crisis resources	4.13 (0.96)
Rewards (for medication adherence, attendance in treatment or abstinence from opioids)	4.11 (0.92)
Coping skills education and practice	4.09 (0.88)
Medication reminders	3.87 (0.92)
Motivational messages	3.85 (0.99)
Providing feedback on progress toward goals	3.87 (1.04)
Information on dealing with stigma, shame, and guilt	3.78 (0.96)
Psychoeducation on co-occurring mental health issues	3.78 (1.07)
Mutual help locator	3.78 (0.99)
Mindfulness or meditation exercises	3.73 (1.03)
Tracking (mood, substance use cravings, and medication adherence)	3.56 (1.07)
Reproductive health information	3.53 (0.98)
Education on medication treatment for opioid use disorder	3.47 (1.15)
General health and wellness tips	3.25 (0.97)

### Phase 2: Development

#### Design Overview

Building on our prior work [23], we used the information from the women with lived experience and provider qualitative interviews, as well as the provider survey data to develop the *Healthy Women* intervention, which includes a web-based component and a mobile component. Content was reviewed by experts in treating opioid use disorder, gender-specific treatment for women, and digital therapeutics (RDW, SFG, and ANCC), and revisions were made to the digital intervention. Finally, the web-based component was beta-tested with 5 participants from the pilot trial and further refined based on their feedback.

#### Web-Based Component

The web-based component is directly adapted from our gender-specific digital intervention for women with SUDs [23], which used material from the WRG [5,6,22]. It was developed in REDCap and contains six topic modules: (1) opioids and treatment, (2) the effects of alcohol and other drugs on women’s health, (3) navigating everyday life without using substances, (4) women and relationships, (5) violence and abuse: getting help, and (6) managing co-occurring mental health problems. The *opioids and treatment* module was developed specifically for this study based on information obtained from the qualitative interviews and survey data, as well as publicly available information from the National Institute on Drug Abuse [27] and the Substance Abuse and Mental Health Services Administration [28]. The other 5 topics were derived from the WRG and our previous work [23] and were modified based on the interview and survey data (Table 6). Topics are presented through a mix of infographics, text, videos, and interactive questions. Each topic starts with an introduction to the topic and ends with take-home messages reviewing the main points. Participants engage with the web-based component first, and it is expected to take 45‐60 minutes to get through all 6 topics. Table 6 shows the content for each of the 6 modules and the associated themes and codes from the qualitative interviews that informed the content.

**Table 6.** Components of web-based modules as informed by qualitative data.

Module names and content, and associated themes and codes	Interview source		Illustrative quotes
	Women with lived experience	Providers	
**Opioids and treatment** **Gender differences in opioid use** **Treatment for OUD** ^**a**^ **Education about MOUD** ^**b**^ **Myths and facts about MOUD** **MOUD during pregnancy**			
Negative beliefs or stigma about MOUD	✓	✓	“I was against [Suboxone]. I didn’t believe in it. If you have to wake up every day and take something, you might as well get high.” (woman with lived experience) “They’re going to get a conflicting message elsewhere that says no you shouldn’t be on medication because if you are on medication then you’re not in recovery.” (provider)
MOUD-specific barriers	✓	✓	“They were giving me a high dose [of methadone], and I was really sleepy, and I felt like I was dopey, like I was a drug addict again.” (woman with lived experience) “[T]hat is a more complicated discussion about staying on it versus not staying on it... Do you go off the thing that helped you become so stable?” (provider)
Family responsibilities	✓	✓	“Because I’d like to have children, and—you know, I don’t want them to be born, like, addicted to drugs.” (woman with lived experience) “I do wonder about concerns about medication and pregnancy...in general [it’s] a concern of women of childbearing age, though, certainly we know that being on medication-assisted treatment is safer than using illicit substances or going through withdrawal while pregnant.” (provider)
**Effects of alcohol and other drugs on women’s health:** **Education on polysubstance use** **The effects of alcohol, cannabis, stimulants, and tobacco on women’s health** **Self-care strategies**			
Polysubstance use	✓		“My drug of choice is crack. And that’s what I would use first. Then I would use the opiates to come down.” (woman with lived experience)
Self-care	✓		“I’ve really been working on that here in this program trying to focus on me more and not so much on every different thing... So just kind of take one thing at a time, and I’m trying to learn how to love myself again. Because I’ve really lost that along the way.” (woman with lived experience)
**Navigating everyday life without using substances:** **Identifying and managing triggers** **Avoiding high-risk situations** **Refusing substances** **Disclosure** **Having fun without using substances**			
Coping skills	✓		“I need coping mechanisms so I don’t use drugs with people.” (woman with lived experience) “It’s just blocking people from being able to reach me...and I’m letting people know, ‘Listen, I’m clean. Stay away from me.’" (woman with lived experience)
Negative beliefs about SUD^c^ treatment	✓		“I was always against treatment. I never agreed with taking someone out of their environment, patching them up and then putting them back in their environment... I felt like, ‘Why can’t you teach me how to live and be in recovery in my environment, because that’s where I’m going back to?’” (woman with lived experience)
Negative beliefs about SUD treatment	✓		“When I usually relapse is during my menstrual [cycle].” (woman with lived experience)
**Women and relationships:** **Positive and negative effects of partners on recovery** **Peer support** **LGBTQ+**^**d**^ **resources**			
Partners/relationships	✓	✓	“If I go back to [my ex-girlfriend], I know I’ll go back to using.” (woman with lived experience) “It’s happened at least once or twice that the woman doesn’t want to go onto like a replacement type therapy an agonist treatment if the partners not going to be on it.” (provider)
Support from others/social network	✓	✓	“...if the people around you are supportive. Because that’s a very rare thing in recovery for females and stuff... A lot of people have already turned their backs on us. So if we do have support, we need it.” (woman with lived experience) “I think family support, family psychoeducation, as well as helping the individual rebuilding sober supports.” (provider)
**Violence and abuse: getting help** **Education on types of abuse** **Examination of past or current relationship** **Safety planning** **Domestic violence resources**			
Trauma and abuse	✓	✓	“The trauma that we face out there in the streets is much different for females than it is for males. We’re more vulnerable and we’re more easily preyed on than the men are. So it’s much more dangerous for us on the street than it is men.” (woman with lived experience) “Women are more subject to intimate partner violence and everything else connected to that.” (provider)
**Managing co-occurring mental health problems:** **The connection between substance use and depression, anxiety, and trauma** **Coping strategies to manage stress**			
Co-occurring disorders	✓	✓	“The depression and things are important. If I can get that under wraps, then maybe my using would not take over my life like that.” (woman with lived experience) “With women with co-occurring psychiatric disorders—which that’s prominent—I think that receiving treatment for more than just the opioid use disorder is an important piece of maintaining adherence with the medication.” (provider)
Trauma	✓	✓	“Past traumas probably should be worked on. Because if you’re using, I feel like you’re already going through enough. You’re probably going to be in the streets and going through stuff.” (woman with lived experience) “The women have this ongoing trauma of different kinds. So they have multiple ACEs [adverse childhood experiences], and then multiple things going on in adulthood that continue.” (provider)
Coping skills	✓		“I want to learn more coping skills because I can’t keep running away to detox when I want to stop. I have to learn how to deal with it on my own.” (woman with lived experience)

a OUD: opioid use disorder.

b MOUD: medication treatment for opioid use disorder.

c SUD: substance use disorder.

d LGBTQ+: lesbian, gay, bisexual, transgender/transsexual, queer, and other minority sexual orientations and gender identities.

#### Mobile Component

**Figure 2.**
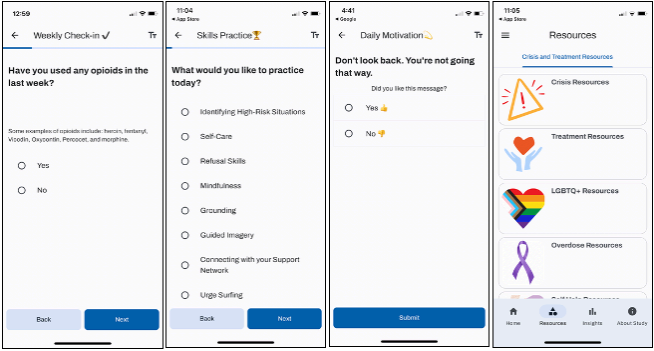
Mobile components of the *Healthy Women* intervention.

The mobile component (Figure 2) of the intervention was developed using Catalyst (MetricWire Inc) and consists of 4 parts: (1) motivational messages, (2) skills practice activities, (3) weekly check-ins, and (4) resources. These features were selected based on the data from the qualitative interviews. Participants have access to the mobile component for 12 weeks.

*Motivational messages* are sent daily. These messages are derived from online searches of inspirational content related to recovery and women. In addition, inspirational messages shared by qualitative interview participants are also included. Participants can “like” or “dislike” the motivational message each day, which allows us to gather data for future refinement of the mobile component.

Participants can choose from 10 different *Skills Practice* activities: (1) identifying high-risk situations, (2) self-care, (3) refusal skills, (4) mindfulness, (5) grounding, (6) guided imagery, (7) connecting with your support network, (8) urge surfing, (9) challenging negative thoughts, and (10) combating self-stigma. Skills practice activities were chosen to address key themes from the qualitative interviews, and many were derived from skills practice activities in the WRG [22]. Participants receive notifications to complete skills practices twice a week. They are encouraged to try each skill once but are not required to complete all of the skills. The guided imagery and mindfulness skills are taught through audio recordings, while the other 8 skills use branching logic to help participants learn the skills in an interactive way.

*Weekly check-ins* consist of questions about participants’ weekly use of opioids and their adherence to MOUD. Participants who report no use of opioids and/or compliance with medication receive positive feedback. Participants who report opioid use and/or noncompliance with medication receive encouragement to focus on their recovery.

Participants can access the *Resources* tab any time they log in to the app. Resources include crisis resources, treatment finder information, overdose prevention information, mutual help group finders, and lesbian, gay, bisexual, transgender/transsexual, queer, and other minority sexual orientations and gender identities resources.

#### Expert Review

Content for the web-based and mobile components was reviewed independently by RDW, SFG, and ANCC, who have expertise in treating opioid use disorder, gender-specific treatment for women, and digital therapeutics. Comments were compiled, and the principal investigator (DES) discussed any discrepant comments with the reviewers to reach a resolution. Multimedia Appendix 3 provides a full list of changes that were suggested by reviewers.

The changes suggested by reviewers and implemented were categorized into four types: formatting, rephrasing, content additions, and content removal. Formatting changes included reordering content for consistency and clarity, breaking up information to reduce cognitive load, and changing the visual color scheme to improve readability. Rephrasing changes had three purposes: to improve clarity, to simplify language (eg, reduce academic language and lower reading level of words), and to increase the accuracy of the content. Two content additions were recommended and implemented: (1) to add information on the effects of xylazine and a warning that it may be mixed into other illicit drugs and (2) to add Alcoholics Anonymous to the list of mutual help groups. Content that was removed included guidelines for alcohol use, 1 of the true or false questions that reviewers felt was confusing, and an example that was deemed too general.

There were only 2 discrepant comments that needed to be resolved. One reviewer suggested giving participants the option to skip the section that provides information on pregnancy and MOUD, as it may not be applicable to some participants (eg, individuals who are beyond child-bearing age or unable to conceive). However, the 2 other reviewers felt that this information could be important for all participants, as there are many misconceptions about MOUD during pregnancy, which can contribute to stigma against women taking MOUD when pregnant. Therefore, it was decided not to allow participants to skip this section. The other discrepant comment was around presenting information on dietary recommendations for alcohol consumption. One reviewer recommended defining standard drinks to increase understanding of the drinking guidelines. However, another reviewer recommended removing the guidelines entirely and replacing them with information describing the importance of abstaining from alcohol while in recovery from opioids. This change was agreed upon by reviewers as it was noted that the alcohol guidelines are meant for individuals without a history of SUD.

#### Beta-Testing Results

The mean age of the beta-test participants was 33.4 (SD 8.5, range 24‐42 y) years; all participants identified as White and non-Hispanic or Latina. Four of the 5 participants were unhoused or in temporary housing at the time of study enrollment (Multimedia Appendix 4 for additional demographic characteristics for the beta-testing sample). Four participants were initiated on methadone, and 1 participant was initiated on buprenorphine in the past 30 days.

A full list of participant comments is included in Multimedia Appendix 5. Two participants commented that they liked the interactive true or false questions, and 3 described appreciating the women-focused information. Three participants expressed concerns that there was too much text, which may be hard for individuals in early recovery. Therefore, we went through each module and replaced text with infographics whenever possible. We also reorganized some of the information based on participants’ comments, such as adding take-home messages not only at the end of the modules but also at the beginning of each module and providing examples of triggers before asking participants to list their own triggers. One participant felt that the gender-specific information on substance use risk could give women an excuse to keep using substances; however, another participant felt that it was helpful to learn about gender-specific risks. Given that we received contradictory feedback on this issue, we decided not to make this change. Finally, 1 participant felt that the feedback given to individuals who report both positive and negative influences of a partner could be more directive on how to deal with this situation. After discussion with the study team, it was decided to keep the original wording as it was designed to help women think through the possible ways this type of partner could affect their recovery.

## Discussion

### Principal Findings

This study outlines the process for developing the *Healthy Women* intervention aimed at increasing women’s engagement in MOUD. The multiphase, mixed methods study included qualitative interviews with women with lived experience of OUD, qualitative interviews with OUD treatment providers, a survey of OUD treatment providers, expert review of the content, and feedback from end-users.

Results from phase 1 identified important topics for engaging women in MOUD treatment, as well as features and content to include in a digital intervention. Several of the barriers to treatment and important factors to address in treatment have been identified in previous reviews of women and SUDs [5,29,30]. However, this study extends the literature by identifying factors specific to women with OUD, including family responsibilities, MOUD-specific barriers, stigma associated with MOUD, and factors motivating MOUD initiation (eg, pregnancy). Although some of these could be endorsed by men as well, women in this study often described the additional burdens on women and societal stigma toward women with OUD. This study also extends the current literature by providing information on features that would be most helpful to include in technology-based tools for women with OUD.

Data from qualitative interviews also revealed barriers to technology use in this population, which could be helpful in developing successful implementation strategies to deploy these tools to women who need them. One of the barriers to using technology as part of treatment was the lack of access to mobile phones. Although studies have shown high rates of smartphone ownership among individuals with SUD [31,32], participants in this study described inconsistent access to their phones. Individuals with SUDs are disproportionately affected by financial and housing instability, which can limit their access to smartphones and reliable data plans. Although this was identified as a significant barrier during the formative phase, the pilot trial of the *Healthy Women* intervention was not initially funded to provide phones to research participants. However, we were eventually able to secure additional funds to provide smartphones to participants, inclusive of a 4-month data plan, which was implemented after 18 participants had been enrolled. Participants are allowed to keep the phone after they complete the study. In clinical settings, digital navigators could be useful in helping patients identify options for affordable or free smartphone plans [33].

During phase 2, common themes and suggested features from the qualitative interviews and survey data were incorporated into a digital platform to create the *Healthy Women* intervention. The intervention was then reviewed by experts, who recommended additional changes to the formatting, wording, and content. Finally, additional changes were recommended by end-users to the web-based model during the beta-testing phase. Changes made in response to end-user feedback during beta testing centered on reducing the amount of text through additional infographics and reorganizing text to improve clarity and emphasize important points. A randomized controlled pilot study (phase 3) is currently underway to examine feasibility, satisfaction, and engagement with the digital intervention, as well as to collect preliminary estimates on the effect of the intervention on engagement in MOUD.

### Limitations

Although we were able to incorporate many of the suggested features into the design of the *Healthy Women* intervention, some suggestions were out of scope for this initial version, such as connecting to peers and providers through the app, providing appointment reminders, and in-app rewards. There were several system-level barriers identified by women with lived experience and providers, which were also confirmed by the survey data. Examples of these system-level barriers included a lack of treatment beds available for women, legal issues and involvement of the Department of Children and Families, and restrictive clinic rules (eg, having to go daily to get methadone during a restricted time frame). These were out of the scope of this study but are important factors to address as part of comprehensive treatment for women with OUD. Another limitation was the lack of racial and ethnic diversity in the beta-testing sample, which may limit the generalizability of the findings. In addition, although research assistants tested the mobile component for functionality prior to launching the study, we were unable to beta-test the mobile component of the intervention with end users due to limitations on the study timeline and funding. However, data collected from the pilot trial will provide important information and feedback from end-users on the mobile component. For this study, we used separate platforms (ie, REDCap for the web-based component and MetricWire for the mobile component), as these were available to the research team and cost-effective. Ideally, the web-based components and the mobile app would be built in the same platform. If shown to be effective, future versions of the *Healthy Women* intervention could be built in an integrated platform with the potential for sustainability and broader scale-up.

### Conclusions

Medication treatment is the gold standard of care for individuals with OUD, yet retention rates are low. Thus, it is important to develop sustainable strategies to engage individuals early in MOUD treatment. Gender-specific treatment for women has been associated with increased engagement, greater satisfaction with treatment, and improved treatment outcomes [4-7]. The *Healthy Women* intervention was developed to address the unique needs of women with OUD to increase their engagement in MOUD treatment. Data collected from the pilot trial, which is currently underway, will provide important information on the feasibility and preliminary effectiveness of this intervention.

## Supplementary material

10.2196/85195 Multimedia Appendix 1 Women with lived experience interview questions.

10.2196/85195 Multimedia Appendix 2 Provider interview questions.

10.2196/85195 Multimedia Appendix 3 Feedback from expert reviewers.

10.2196/85195 Multimedia Appendix 4 Demographic characteristics of beta test participants.

10.2196/85195 Multimedia Appendix 5 Notes from beta testing participants’ responses.

## Abbreviations

ADATP: Alcohol, Drug, and Addiction Treatment Program
DDBT: Discover, Design and Build, and Test
IRB: Institutional Review Board
MOUD: medication treatment for opioid use disorder
OUD: opioid use disorder
REDCap: Research Electronic Data Capture
SUD: substance use disorder
WRG: Women’s Recovery Group
