# Supplementary material for: Development of the Healthy Women Intervention to Increase Women’s Engagement in Medication Treatment for Opioid Use Disorder: Mixed Methods, User-Centered Design Approach
Source: JMIR Form Res. 2026 Mar 31;10:e85195. doi: 10.2196/85195 (PMC13037578; doi:10.2196/85195)
Supplement: Multimedia Appendix 1 [file formative-v10-e85195-s001.docx]

**Interview Questions for Women with Lived Experience of Opioid Use Disorder**

*“Thank you very much for participating in this focus group. I really appreciate you taking the time today to meet with us. I will be asking you a series of questions about your experiences with treatment. This information will be very helpful for better understanding women’s experiences in treatment for opioid use disorder in order to better enhance our treatment services. We will meet for approximately an hour and 15 minutes and this focus group will be audiotaped. Please remember that anything you say or hear here should be kept confidential. Thanks again for your participation.”*

1. What types of treatment are you engaged in for your opioid use disorder?
   1. Examples: group therapy, individual counseling, medication treatment.
2. What topics do you think would be particularly important to address as part of your treatment?
   1. Are there certain topics that are particularly important for you as a woman?
3. What are your thoughts on medication treatment for opioid use disorder, in general?
   1. What are your thoughts on specific medications:
      1. Burpenorphine (Suboxone)
      2. Injectable Naltrexone (Vivatrol)
      3. Methadone
4. For those of you who are interested in medication treatment, are there any barriers that get in the way of you accessing it?
   1. Barriers specific to women?
5. For those of you not interested in receiving medication treatment, what are your main reasons?
6. For those unsure, what might lead you to decide to take medications or to not take medication?
   1. Do you think there are issues that you as a woman face (that men might not) that would affect your decision?
7. How interested would you be in using technology, such as a mobile app that you would download your phone, as a way to stay engaged with your treatment goals?
   1. How about to stay on track with medication treatment?
8. For those who would use a mobile app, what features would you most want to see in a mobile app? For example, reminder messages, motivational/inspirational messages, progress tracking, calendar, videos, meditations through words or music, etc.
9. What might get in the way of you using a mobile app?

*If need prompts: Internet access? Data usage? Cost? Smartphone access? Concerns someone might see it? Other?*

1. How could technology help you communicate issues to your treatment providers?
   1. Would you use a program that gave feedback to your treatment provider on your progress?
      1. If yes, what type of information would you want to share with your provider?
   2. What concerns might you have about confidentiality of the data that is collected?
