# Supplementary material for: Development of the Healthy Women Intervention to Increase Women’s Engagement in Medication Treatment for Opioid Use Disorder: Mixed Methods, User-Centered Design Approach
Source: JMIR Form Res. 2026 Mar 31;10:e85195. doi: 10.2196/85195 (PMC13037578; doi:10.2196/85195)
Supplement: Multimedia Appendix 2 [file formative-v10-e85195-s002.docx]

**Provider Interview Questions**

*“Thank you very much for participating in this interview. I really appreciate you taking the time today to meet with us. I will be asking you a series of questions about your experiences treating women with opioid use disorder. This information will be very helpful to better understand women’s experiences in treatment for opioid use disorder in order to enhance our treatment services.”*

1. What types of treatment do you provide for patients with opioid use disorder?
   1. Examples: group therapy, individual counseling, medication treatment.
   2. How many years have you been working with individuals with OUD?
2. What are the most common reasons that people choose to initiate MOUD?
   1. Are there reasons specific or more important to women?
3. What are the main reasons that people decline MOUD?
   1. Do you notice specific reasons that are more often cited by women?
4. What do you think are the barriers that get in the way of individuals initiating medication treatment for opioid use disorder?
   1. Are there barriers that are specific to women or more salient for women?
5. For individuals who successfully initiate MOUD, what factors contribute to sustained use of MOUD?
   1. Are there factors that are particularly important for women in supporting their sustained use?
6. What are the main difficulties that individuals with OUD face after initiation on MOUD that interferes with their staying on medication?
   1. Any of these factors more common or specific to women?
7. For people who are struggling to stay on track, what would help them stay on track with medication treatment? Follow-up with specific to women?
8. Do you see a significant role for technology (e.g., mobile app) in helping people initiate or stay on MOUD?
   1. Do you see that being different for men and women?
9. If your patient was using a mobile app to help her stay on track with treatment goals, would you want to be able to see her progress (i.e., have access to app data)?
   1. What type of information would be useful for patients to share with you from an app?
