# Supplementary material for: Development of the Healthy Women Intervention to Increase Women’s Engagement in Medication Treatment for Opioid Use Disorder: Mixed Methods, User-Centered Design Approach
Source: JMIR Form Res. 2026 Mar 31;10:e85195. doi: 10.2196/85195 (PMC13037578; doi:10.2196/85195)
Supplement: Multimedia Appendix 3 [file formative-v10-e85195-s003.docx]

**FEEDBACK FROM EXPERT REVIEWERS**

**Changes to web-based modules suggested by reviewers**

***Formatting***

- Change overall font color scheme to blue and green tones, rather than yellow and red to improve readability
- Make the “MYTH/FACT” and “TRUE/FALSE” options consistent so the responses are in the same order
- Break up information on naloxone, fentanyl, and xylazine into smaller sections/less text
- Replace the telescope picture with person-centered picture
- Basic facts about cannabis: describe THC before CBD
- Fix the spacing of the bullets under “common symptoms of anxiety”

***Rephrasing***

***To improve clarity***

- For the true/false questions update all so that they read “sorry, ___” for the wrong and “you’re correct, __” for right because “yes, it’s false” and like “no, it’s true” may get confusing especially if the statement already has a negative word in it
- “Be thoughtful of environment” – not sure what this means, may need to simplify/reword
- Women and relationships learning objectives: Add “and recovery” to the end of #1. Change #2 to say: “Strategies that will help enable you to have and maintain healthy relationships.”

***To simplify language***

- Replace ‘thank you for completing…” with something less formal and more congratulatory
- Remove sentence that names the ‘telescoping’ process at the end “We call this process…”
- “Negative feelings stemming from…” – reword to a more conversational tone
- For the nicotine True/False question: change “hypertension” to “high blood pressure”
- change “interpersonal conflict” to “conflict with others”
- change “Insomnia/lack of sleep” to “sleep problems”
- Change bullet 3 of take home messages to say: “Women may experience a quicker progression of their problems with substance use compared to men.”
- Reword absence of a partner to “not having a partner”

***For content accuracy***

- Change the word “doctor” to “prescriber”
- Change header from “Effects of Alcohol and Drugs on Women’s Health to “Effects of Other Drugs and Alcohol on Women’s Health”
- For slide that says “In the following pages…” change text to: “In the following pages when we mention cannabis, we are specifically referring to THC, not CBD. Please answer the following questions with THC in mind.”
- For the answers to the myth about cannabis being addictive. Change answers to say “Cannabis can be addictive…” instead of “Cannabis is addictive…”
- The information on cocaine leading to infertility may not be needed – I know there is some data on this, but it may feel like a scare tactic – consider rephrasing to say that it disrupts women’s hormones and cycles and can lead to problems getting pregnant
- Reword relationship sentence to say to say “partners can influence substance use positively or negatively” instead of “positively and negatively”
- MOUD pictures – remove the brand names from the methadone box

***Content additions***

- Add information on Xylazine awareness
- Ways to connect w/ others: Add AA to where it says NA and SMART recovery
- Define standard drinks in the alcohol guidelines section *- this change was NOT implemented; upon discussion with other reviewers there was consensus that the message should be around abstaining from alcohol, rather than presenting recommended dietary guidelines for alcohol consumption which are meant for individuals without history of substance use disorder*

***Content removal***

- Take out alcohol guidelines slide, instead add text: “Even when not using opioids, even small amounts of alcohol can lead women to make risky decisions that they might not make when they are sober, such as, returning to opioid use.”
- Remove this True/False question: Prescription stimulants, like Adderall and Ritalin, are not addictive.
- Remove self-care from examples (too general)
- Allow participants to skip pregnancy and MOUD info – *this change was NOT implemented, as 2 reviewers disagreed with this suggestion*

**Changes to mobile component suggested by reviewers:**

***Content additions***

- Weekly check-in questions: add Brixadi® to list of buprenorphine brand names
- Add 4 additional skills to skills practice exercises (connecting with your support network, urge surfing, challenging negative thoughts, combatting self-stigma) for a total of 10 skills exercises
- Add overdose prevention resources/information to Resources section
- Add links for mutual help groups to Resources section
