# Supplementary material for: Development of the Healthy Women Intervention to Increase Women’s Engagement in Medication Treatment for Opioid Use Disorder: Mixed Methods, User-Centered Design Approach
Source: JMIR Form Res. 2026 Mar 31;10:e85195. doi: 10.2196/85195 (PMC13037578; doi:10.2196/85195)
Supplement: Multimedia Appendix 4 [file formative-v10-e85195-s004.docx]

#### **Demographic Characteristics of Beta-Test Participants (N=5)**

| **Age (years), M (SD)**  Range | 33.4 (SD = 8.5)  24 - 42 |
| --- | --- |
| **Race*, n (%)**  White | 5 (100.0) |
| **Ethnicity, n (%)**  Hispanic or Latina | 0 (0.0) |
| **Sexual orientation, n (%)**  Heterosexual/straight  Bisexual | 2 (40.0)  3 (60.0) |
| **Marital status, n (%)**  Never married  Separated | 4 (80.0)  1 (20.0) |
| **Education, n (%)**  Less than high school  High school graduate/GED  College graduate | 1 (20.0)  3 (60.0)  1 (20.0) |
| **Primary residence, n (%)**  Apartment/house  Unhoused (Outdoors/street, abandoned or public building, automobile)  Temporary housing | 1 (20.0)  2 (40.0)  2 (40.0) |
| **Occupational status, n (%)**  Unemployed  Disability | 2 (40.0)  3 (60.0) |
| **Have children, n (%)**  How many? M (SD) | 3 (60.0)  2.3 (1.2) |
