# Supplementary material for: Development of the Healthy Women Intervention to Increase Women’s Engagement in Medication Treatment for Opioid Use Disorder: Mixed Methods, User-Centered Design Approach
Source: JMIR Form Res. 2026 Mar 31;10:e85195. doi: 10.2196/85195 (PMC13037578; doi:10.2196/85195)
Supplement: Multimedia Appendix 5 [file formative-v10-e85195-s005.docx]

**Notes from Beta-Testing Participants Responses**

**Participant ID: P001**

- “I wouldn’t have known that” in response a true or false question
- Very interested in the effects on miscarriages/ability to get pregnant
- “by telling people that it’s more likely for women to develop a problem than men, it’s giving another excuse for people to use to keep getting high”
- “My neck hurts” afterwards
- She likes the true/false questions because at the ends it tells you why it’s incorrect
- A lot of women that use can’t sit through a long amount of time with lots of info, it’s hard to process completely
- It might be frustrating – maybe more pictures with questions or less information with questions
- Visually it was nice, but add more pictures

**Participant ID: P005**

- “This seems good talking about women and relationships”, related it back to the women’s group
- “I found it interesting to know that women were at a higher risk than men”
- There wasn’t anything that I didn’t really like
- The domestic violence part was helpful as she said she has been a woman who has recovered from that
- Participant indicated she would like more information on general educational facts of opioids, damage it can cause, and more about the women/men difference
- Perfectly balanced with questions
- She said it got a little confusing, but that was normal for her because she has a learning disability
- She didn’t think it was overwhelming or too much at all.

**Participant ID: P006**

- She said a lot of the things she was asked/learned about women vs men she assumed were true but it was good to be confirmed
- She liked it
- She has done a lot of CBT and DBT day programs using worksheets, so she said a lot of this program reminded her of that and was helpful

**Participant ID: P008**

- Looks really pretty, nice to look at, thinks that there is too much information on one page (too many slides on one page)
- Take home messages are concise, not in alignment with the rest of the information. Participant said that the take home messages are summarizing the information well, but seems like participant wishes the rest of the information was also more concise.
- Thinks maybe it would be helpful to start with the take home messages and then provide context for them throughout
- Take home messages felt like a breath of fresh air after reading the whole module, maybe would have been good to start with them to set you up for what you’re getting into
- Take home messages are clear and concise and info leading up to it is not (repeat of previous comment)
  - Start with take home messages
- Participant notes being “braindead” right now because she has just been in detox treatment for the last 5 days
- Women and relationships started off with a better intro into what we are going to cover, and she liked that. Says she likes the set-up of this section in general
  - Notes that the other sections seemed to just jump in quickly rather than giving an overview
- Answer to how to approach the situation of having the person score “directly in the middle” (women and relationships question) a bit more thought out. Thinks that this situation is probably very common and it would be helpful to have a more thought-out response on how to address this.
  - This is the question where you answer questions and it gives a response based on how many “yes” and “no” on whether that person is positively or negatively impacting
  - This is referring to M3 Women and Relationships: "Examine your past and present relationships by thinking of an important person in your life..." followed by 6 yes/no questions. If they answer 3 yes and 3 no, they get a response "*It looks like this person has both negative and positive influences on your substance use. You might want to consider what changes can be made to stay on track with your recovery goals. For example, if your partner uses substances, are they willing to give up their own drug use? If you live with your partner, are they willing to make your home an alcohol and drug free zone? Is your partner willing to engage in treatment with you or on their own?*”

**Participant ID: P009**

- She really likes the true/false questions – they feel validating in her experience and knowledge. Participant says that she often doesn’t feel very smart and it feels good to be affirmed that she is knowledgeable.
- “It’s hard to press the buttons” regarding using the iPad for answering questions. Says a stylus would be helpful
- Last question was about triggers, the slide with “here are some triggers that other women with opioid use disorder reported:”, participant thinks that putting this slide before the question asking her to list her own triggers would have been helpful to give some examples of what to say. She was having trouble finding the words to describe what she wanted to express as her own triggers, so having this slide first would have been helpful (and would maybe relieve a bit of frustration trying to find the right words)
- Repeats again after continuing through the module that the information first would be helpful before the questions (still on triggers section)
